# A Winter in Egypt
*"A Winter on the Nile, in Egypt, and in Nubia." By the Rev. Charles D. Bell, D.D., Honorary Canon of Carlisle; Rector of Cheltenham; Author of "Gleanings from a Tour in Palestine and the East." London: Hodder and Stoughton, 27, Paternoster Row. Price six shillings.


**Published:** 1889-02-02

**Authors:** 


### A Winter in Egypt.*

Egypt is the Cleopatra of countries, fascinating in her old age. That long strip of land which the Nile makes fertile in the midst of the surrounding desert, is so full of memories that every inch of it has a voice to charm us. It is the cradle of civilisation; the arts and sciences of to-day were born inEgypt —were born and died so far as the land of their origin was concerned, while they were being handed down to us by Greeks and Romans and Saracens. To the imagination every statue and obelisk, every pillar of its magnificent temples, every stone of its pyramids, is vocal as Memnon, mysterious as the Sphinx. It is a hieroglyph; we unravel the meaning of some of the tokens that tell of bygone learning and culture, but we feel that more remains uncomprehended, the food for dreams and conjectures. Egypt teems with memories of all ages: the Pharaohs—he who protected Joseph and he whom Moses defied; the Ptolemies; Cæsar and Antony, and subtle Cleopatra carrying her smiles and serpentine caresses from one to the other; Hypatia, standing fearless before the monks who thirst to murder her; the fierce Caliphs who destroyed the corrupt Christianity that disgraced the name of its Founder; soldiers of every nation and creed whose blood has enriched its soil, down to the good English blood poured out so short a time ago by Gordon, Hicks, and others, for whom the tears are not yet dried.

Everyone who goes to Egypt must return so full of its charms and wonders that he longs to speak of them. Dr. Bell went to Egypt, and yielded to this temptation—in print. His experiences are simply the pleasant reminiscences of the ordinary visitor. He went as a "Cook's tourist," and makes a good plea for that much-laughed-at firm. If Messrs. Cook do take 'Enery and 'Arriet to Venice to make their Cockney puns upon the Bridge of Sighs, they at the same time open up lands unseen, but not unknown, to many whose culture exceeds their means. The opportunities of education afforded by travel are unrivalled, and something better than laughter is deserved by the enterprising firm who put such good schooling within reach of the multitude. Perhaps Messrs. Cook are not the least of our benefactors.

As we have said, there is nothing remarkable in Dr. Bell's experiences. Alexandria, Cairo (becoming Frenchified in its old age), Luxor, Philæ, and Heliopolis have all been described before: but there is no doubt that the author felt their fascination to the full. One suggestion which he makes we think well worthy of attention. As the French Government subsidise schools in which their language is taught, it would be well, considering the close connexion existing between England and Egypt, to encourage the teaching of English in the schools by some similar means. He also tells an anecdote, new to us, of Ergammum, a king of Nubia. It had been the custom of the priests to indicate to their monarch the time when the gods invited him to join them in the happy realms above. Ergammum, however, had a mind of his own; so, when the summons came to him, he went to the temple in all the state with which the kings were wont to march to their doom; but, arrived there, instead of committing suicide, as he ought to have done, he killed the priests, and went home to his palace. So that tradition came to an end in Nubia.

Dr. Bell's criticism of the legendary portrait of Cleopatra strikes us as singularly infelicitous. He says her aspect is voluptuous and sensual, and more of an oriental than a western style of beauty. What did he expect? He surely did not expect to find in the "serpent of old Nile" anything similar to a rosy, innocent English girl. It is natural that an author who writes "D.D." after his name should take an interest in the various creeds of the countries he visits; but Dr. Bell does not give us sufficient proof of having bestowed real and unbiassed study on Mohammedanism to justify him in his sweeping condemnation of it. Such a sentence as "their prayers are entirely a matter of vain repetition" is quite unjustifiable. A creed which justifies polygamy must, of course, be abhorrent in our eyes, but our utmost disapproval of the religion of Islam should not make us forget what an enormous step it was, in its strict monotheism, from the confused idolatry and social corruption in which Mohammed was born.

* "A Winter on the Nile, in Egypt, and in Nubia." By the Rev. Charles D. Bell, D.D., Honorary Canon of Carlisle; Rector of Cheltenham; Author of "Gleanings from a Tour in Palestine and the East." London: Hodder and Stoughton, 27, Paternoster Row. Price six shillings.

